# Brief Report: Specificity of Interpersonal Synchrony Deficits to Autism Spectrum Disorder and Its Potential for Digitally Assisted Diagnostics

**DOI:** 10.1007/s10803-021-05194-3

**Published:** 2021-07-31

**Authors:** Jana Christina Koehler, Alexandra Livia Georgescu, Johanna Weiske, Moritz Spangemacher, Lana Burghof, Peter Falkai, Nikolaos Koutsouleris, Wolfgang Tschacher, Kai Vogeley, Christine M. Falter-Wagner

**Affiliations:** 1 Department of Psychiatry and Psychotherapy, Medical Faculty, LMU Munich, Nussbaumstrasse 7, Munich, Germany; 2 Department of Psychiatry, Faculty of Medicine and University Hospital Cologne, University of Cologne, Cologne, Germany; 3 University Hospital of Psychiatry and Psychotherapy, University of Bern, Bern, Switzerland; 4 Institute of Neuroscience and Medicine, Cognitive Neuroscience (INM-3), Research Centre Juelich, Juelich, Germany; 5 Department of Psychology, Institute of Psychiatry, Psychology and Neuroscience, King’s College London, London, UK; 6 Department of Psychology, University of Cologne, Cologne, Germany

**Keywords:** Autism spectrum disorder, Interpersonal synchrony, Motion energy analysis, Diagnostics, Social interaction

## Abstract

Reliably diagnosing autism spectrum disorders (ASD) in adulthood poses a challenge to clinicians due to the absence of specific diagnostic markers. This study investigated the potential of interpersonal synchrony (IPS), which has been found to be reduced in ASD, to augment the diagnostic process. IPS was objectively assessed in videos of diagnostic interviews in a representative referral population from two specialized autism outpatient clinics. In contrast to the current screening tools that could not reliably differentiate, we found a significant reduction of IPS in interactions with individuals later diagnosed with ASD (n = 16) as opposed to those not receiving a diagnosis (n = 23). While these findings need to be validated in larger samples, they nevertheless underline the potential of digitally-enhanced diagnostic processes for ASD.

**Supplementary Information:**

The online version contains supplementary material available at 10.1007/s10803-021-05194-3.

## Introduction

Nonverbal abnormalities in the social domain have been identified as a key impairment in autism spectrum disorder (ASD; American Psychiatric Association, 2013). Though being a developmental disorder manifesting throughout the first three years of life, diagnostic services are faced with an increasing demand for diagnostics in adulthood throughout the past years (Murphy et al., 2011), representing a current clinical challenge and one of the ten areas of priority for autism research as published by Autistica (Cusack & Sterry, 2016). Unfortunately, established gold-standard diagnostic tools for children (e.g. ADOS; Lord et al., 2012) are less reliable for diagnosing adults (Maddox et al., 2017) and there is a range of mental health issues associated with social interaction difficulties representing a particular challenge for differential diagnostics in adulthood (Arbeitsgemeinschaft der Wissenschaftlichen Medizinischen Fachgesellschaften/AWMF, 2016). Current popular diagnostic screening tools, such as the Autism Quotient (AQ; Baron-Cohen et al., 2001) or the Empathy Quotient (EQ; Lawrence et al., 2004), aimed at respectively measuring autistic traits and global empathy in a general population, lack specificity in a clinical population (Kästner et al., 2015; Ketelaars et al., 2008). One reason for this might be the marked phenomenological heterogeneity in adulthood, which might be due to several factors, such as development of strategies for compensation of social impairments, termed camouflaging (Lai et al., 2017), and high prevalence of comorbidities (Lai & Baron-Cohen, 2015). The diagnostic process is complex and lengthy, the number of experienced diagnosticians is small, and intervals between referral and first diagnostic appointment are about 6 months (median length for the example of Canada; according to Penner et al., 2018), a situation potentially causing tremendous distress for individuals seeking diagnostic clarification and waiting for clinical psychological treatment. Thus, there is an urgent need for objective diagnostic tools, potentially provided by automatized and objective classification methods (Georgescu et al., 2019).

Though especially adults with ASD without intellectual impairment are prone to social learning and camouflaging their impairments (Lehnhardt et al., 2013), they are nevertheless perceived as somewhat ‘odd’ by peers. For example, Sasson and Morrison (2019) investigated the first impression of adults with ASD vs. typically-developing (TD) adults and found that adults with ASD were rated less favorably than their neurotypical peers on multiple measures, regardless of whether the raters were naïve to the diagnosis or not. Additionally, TD adults have been found to be less willing to further interact with autistic adults (Morrison et al., 2019), strikingly demonstrating the potential social consequences of this bias. Previous research suggests that said awkwardness and reduced connectedness might be due to aberrated coordination of non-verbal behaviors of individuals with ASD with another interactant, a phenomenon that has previously been described as reduced interpersonal synchrony (IPS; Bloch et al., 2019; Georgescu et al., 2020; Kaur et al., 2018; Koehne et al., 2015; McNaughton & Redcay, 2020). Reduced IPS in autistic children with non-autistic adults has been found to correlate negatively with autism symptom severity, regardless of the familiarity of the interactant (Zampella et al., 2020).

Classifying ASD as a disorder of social interaction, multiple attempts have been made to extract diagnostic markers from social interactions. However, surprisingly few studies on IPS have focused on adults with ASD. In a study investigating synchronization in a joint movement game, ASD participants performed worse than TD participants (Brezis et al., 2017). On the other hand, Zapata-Fonseca et al. (2019) found a similar amount of implicit movement coordination in a computer-mediated interaction task, though ASD participants showed less movement variability than their TD counterparts. In a simulated social interaction paradigm participants with ASD and TD were classified with an accuracy of 73% based on their facial expression and vocal parameters when interacting with a pre-recorded actress (Drimalla et al., 2020).

While these findings underscore the potential of social interaction dynamics for digitally assisted diagnostics in ASD, their ecological validity remains an issue. In fact, IPS in ASD has often been investigated in the context of isolated and staged rhythmic motor tasks, such as pendulum-swinging (Fitzpatrick et al., 2016), the rocking chair paradigm (Marsh et al., 2013) or finger tapping (Koehne et al., 2015). Thus, while maintaining highly standardized experimental conditions, generalization of findings to real-life social settings is limited. Particularly, individuals with ASD have pronounced difficulties with real-time social interactions (Redcay et al., 2013) and the IPS deficit in ASD seems to increase for situations with higher social demands (McNaughton & Redcay, 2020). However, research on IPS in naturalistic settings is currently lacking. In a recent study with high ecological validity comparing IPS across different compositions of autistic dyads, we found that IPS was reduced in interactions comprising at least one individual with ASD, regardless of partner diagnosis, suggesting a ‘synchrony signature’ specific for ASD (Georgescu et al., 2020).

In order to assess the potential of this synchrony signature as a diagnostic marker for ASD it needs to be tested for specificity in a real-life clinical setting. This was the aim of the current study. Abnormal IPS has been reported in other psychiatric diagnoses that are associated with social communication difficulties, including schizophrenia (Kupper et al., 2016) and depression (Paulick et al., 2018). While differential diagnostics between schizophrenia and ASD manifests on the basis of presence or absence of so-called positive symptoms (e.g. hallucinations), the differentiation between ASD and other diagnoses presenting with social interaction difficulties is a marked challenge (Lehnhardt et al., 2013). Given the high number of patients seeking ASD diagnosis who are reporting social interaction difficulties but not fulfilling diagnostic criteria, an investigation of the specificity of IPS in a naturalistic setting is therefore essential.

Given any abnormal IPS patterns (Georgescu et al., 2020) might merely be due to a high prevalence of motor difficulties in ASD (Dziuk et al., 2007; Parma & de Marchena, 2016; Vanvuchelen et al., 2007), we additionally assessed dyspraxia in the studied population. Even though motor difficulties do not currently form a part of the diagnostic criteria, recent changes in the Diagnostic Manual for Mental Disorders (DSM-5; American Psychiatric Association, 2013) allow for ASD and developmental coordination disorder (DCD or dyspraxia) to be diagnosed as co-occurring conditions (Caçola et al., 2017). DCD is characterized by significant impairments in performing gross- and fine motor skills, coordination and balance at an age-appropriate level. Though it is suggested that the symptom profiles of each disorder are separable (Caçola et al., 2017), the official recognition as potential comorbidity points towards considerable motor impairments in many individuals with ASD. While approximately 80% of children with ASD are suspected to exhibit pronounced motor difficulties (Green et al., 2009), the body of evidence on autistic adults in this area is surprisingly small. It is suggested that individuals diagnosed with DCD still show symptoms in adulthood, though possibly altered due to interventions in childhood, the development of coping mechanisms or the avoidance of situations where fine-tuned motor skills are necessary (Kirby et al., 2010). First evidence in ASD demonstrates a significantly higher prevalence of dyspraxia in autistic adults than in TD controls (6.9% vs. 0.8%) and these motor impairments have been found to be associated with autistic traits in the general population (Cassidy et al., 2016). Considering this high prevalence and the suggested relationship between early motor difficulties and impaired social functioning in children with ASD, autistic adults should exhibit significantly higher motor impairments than adults without an ASD diagnosis. Importantly, this might in turn hamper IPS between interactants and should therefore be assessed in an analysis of the specificity of IPS to rule out dyspraxia as a potential bias.

Thus, in the current study we employed an observational study design and investigated the specificity of abnormal IPS as a potential diagnostic marker for ASD within real-life clinical populations referred to differential diagnostics in two specialized adult autism clinics. In addition to the standard screening tools, we quantified IPS from videotaped initial diagnostic examinations and measured self-rated symptoms of dyspraxia. Crucially, a diagnostic decision had not been made at the time of data collection and final diagnostic groups were only formed in retrospect, therefore blinding the respective clinician interacting with the advice searching patient.

## Methods

### Participants

Participants were drawn from a representative clinical referral population of adults referred to autism diagnostics to the specialised autism outpatient clinics of the University Hospitals of Munich and Cologne. All participants were assessed while undergoing real-life diagnostic procedures and had been referred to the specialist clinics by medical consultants (psychiatry and neurology) on the basis of suspicion of a possible ASD diagnosis due to reported social-emotional symptoms. Diagnostic procedures have been conducted in accordance to German guidelines for diagnosis of ASD in adulthood (German S3-guidelines; AWMF, 2016) comprising neuropsychological and clinical assessment of at least two independent trained clinicians. The assessed population was retrospectively split into ASD + cases (*n* = 16) consisting of individuals who received a diagnosis of F84.5 (*n* = 10; *Asperger Syndrome*), F84.1 (*n* = 3; *Atypical Autism*), or F84.0 (*n* = 3; *Childhood Autism*) according to ICD-10 (World Health Organization, 2016) and ASD- cases (*n* = 23) consisting of individuals for whom any F84 diagnosis (including F84.9) was ruled out.

The groups were frequency-matched with respect to IQ (*t*(37) =  − 0.437, p = 0.664), motor difficulties (*U* = 239.5, p = 0.116) and age (*U* = 134, p = 0.157). Descriptive statistics can be found in Table 1. All participants gave written informed consent before study participation. The study was approved by the ethics committee of the medical faculties of the LMU Munich and the University of Cologne, in agreement with the Declaration of Helsinki.

**Table 1:** Demographic information

	ASD + (n = 16; 5 female)	ASD − (n = 23; 14 female)	Group comparison (p*-*value)
Age	34.19 ± 12.41	39.57 ± 12.29	0.157
Verbal IQ	103.19 ± 19.48	105.35 ± 11.33	0.664
AQ	36.75 ± 6.76	35.70 ± 8.26	0.830
EQ	16.06 ± 9.98	19.74 ± 11.02	0.294
ADC	54.20 ± 15.19	42.9 ± 19.45	0.116
TAS20	59.44 ± 13.83	64.13 ± 10.24	0.165
BDI	13.53 ± 10.18	19.3 ± 8.63	0.064

Mean values and standard deviations, as well as group comparison (p-value) of age, verbal IQ (‘Wortschatztest/WST’), Autism Quotient (AQ), Empathy Quotient (EQ), Toronto Alexithymia Scale (TAS20), Adult Developmental Coordination Disorder/Dyspraxia Checklist (ADC), Beck’s Depression Inventory (BDI). BDI data for one ASD+ person was missing and was imputed with the group mean. For the dyspraxia questionnaire (ADC), scores of 4 participants (3 ASD− and 1 ASD+) were missing and imputed with the respective group average. For age and TAS20 we performed a Mann–Whitney test because the values in the ASD− group were not normally distributed. For ADC we performed a Mann–Whitney test because the values in the ASD+ group were not normally distributed

### Design and Procedure

Data was collected in an observational study design accompanying standard diagnostic procedures. The first diagnostic interview after an initial admission appointment of every participant was videotaped on a HD camera (Sony HDR-CX625) with a frame rate of 25 fps. The interviews were conducted by eight different diagnosticians (including JK). Subsequently, patients underwent several hours of analysis of medical history, former parental/caretaker interviews and neuropsychological assessments (according to German S3-guidelines; AWMF, 2016). Finally, a diagnostic decision was eventually made by one of three specialized clinical experts (including KV and CFW) who did not take part in the data collection. Participants were post-hoc assigned to either the ASD + or ASD- group according to diagnostic decision.

The video setup, lighting and seating arrangement closely resembled the setup of our previous study on reduced IPS in autism (Georgescu et al., 2020). To ensure comparable filming conditions, stable ambient light was kept and the camera position remained static throughout the interviews. Chairs were positioned in fixed spots at 45° angle from the camera. Seating positions of participant and diagnostician (left, right) were allocated in a counterbalanced order. A video vignette of the first 14 min of each diagnostic session was extracted, to allow for unobstructed analysis with both participants seated and without any external disturbances.

Participants filled out the AQ (Baron-Cohen et al., 2001) to measure autistic trait severity, the EQ (Baron-Cohen & Wheelwright, 2004), an index of global empathy, and the degree of self-rated motor difficulties, as indicated by a translated German version of the Adult Developmental Co-ordination Disorders/Dyspraxia Checklist (ADC; Kirby et al., 2010), and completed a post-test questionnaire to rate the quality of the interaction and the extent by which they felt influenced by the recording camera. As part of the standard clinical assessment, participants additionally completed the German version of the Beck’s Depression Inventory (BDI; Hautzinger et al., 1994), the Systemizing Quotient (SQ; Frith et al., 2003), and the 20-Item-Toronto Alexithymia Scale (TAS20; Bagby et al., 1994).

Videos were analyzed post-hoc with Motion Energy Analysis Version 3.10 (MEA; Ramseyer & Tschacher, 2011), an observer-independent, objective tool to quantify movement. MEA is a frame-differencing method used to quantify the amount of movement present in a video. The software extracts changes in grayscale pixel values frame by frame, so-called motion energy, of videos in pre-defined regions of interest (ROI; Ramseyer & Tschacher, 2014). With stable background and lighting, each change in pixels/motion energy indicates body motion of the participants. In line with previous studies using MEA, two ROIs were chosen manually for each participant, namely head and upper body. MEA then yields movement time series of gray-scale pixel change of every frame for each ROI above a manually-set threshold within the program that allows for the filtering of signal fluctuations as opposed to real movement (for an in-depth description of MEA see Ramseyer et al., 2019). After careful inspection of our data, we chose a value of 15, thereby lowering the default (25) to adjust for our lighting specifics. To compute interpersonal synchrony (IPS), time series were cross-correlated in moving windows of 60 s for all time lags between − 5 and + 5 s, using R custom software (rMEA; Kleinbub & Ramseyer, 2020). Cross-correlations were Fisher’s *Z*-transformed to allow aggregation and their means aggregated in absolute values over the 14-min interval, yielding two interpersonal synchrony values per dyad, for head and body movement respectively. One vignette had to be split in half due to interactants exchanging documents half way through the conversation. The resulting IPS values were averaged across the two vignettes. We have also added the values of the head and body ROI (as they are mutually exclusive) to compute a total ROI per person.

In order to evaluate the significance of synchrony values, we need to validate the procedure against coincidental synchrony (i.e. synchrony that might have occurred by chance). To this end n = 1,000 surrogate synchronies were computed (out of a possible of N = 3,120) by aligning time series of participants in random order who never actually interacted with one another (Ramseyer, 2019). The resulting surrogate datasets were analyzed in the same manner, again yielding two global values per dyad (head and body) for pseudosynchrony.

The relative amount of movement quantity of every participant was calculated as the percentage of frames with above-threshold movement (number of frames with frame differences > 0 divided by total number of frames) within every ROI (Ramseyer & Tschacher, 2014). The values of the respective interactants were averaged within every dyad to obtain an indicator of mean movement quantity per dyad. A table of the descriptive statistics can be found in the supplementary results.

The context and nature of these interactions required that the clinician interacts with objects (a clipboard and pen). This makes the synchrony in the body ROI more difficult to interpret. For reasons of completion we have reported these, along with the total ROI in the supplementary materials. We therefore report the head ROI and have good reason to do so: In previous research on IPS, head synchrony has been associated with the overall outcome of psychotherapy (Ramseyer & Tschacher, 2014) and has been found to be indicative of the symptom profile of schizophrenia (Kupper et al., 2016), a psychiatric disorder that shares several common features with ASD, though the exact dynamic patterns remain to be investigated. Further, head movement dynamics (pitch, yaw and roll) have been found to differ between children with and without ASD in a social context (Martin et al., 2018), deeming them appropriate for further investigation in a synchrony context.

## Re﻿su﻿lts

### IPS vs. Pseudosynchrony

To ascertain that the method yielded valid IPS measurements, we compared mean IPS with surrogate data across participants using the Mann–Whitney test, as a nonparametric alternative for independent samples t-test (Ramseyer, 2020), because a Shapiro–Wilk test showed a significant departure from normality for the pseudo-IPS values (W = 0.991, p < 0.001). In line with previous studies applying MEA, IPS was significantly higher than pseudosynchrony in the head ROI (*U* = 23,354, p = 0.018, one-tailed, *d*_Cohen_ = 0.198).

### Movement Quantity Across Groups

To rule out the overall amount of movement as a confounding factor for potential differences in IPS, we examined differences in average movement quantity per dyad across groups. We performed an independent samples t-test. No significant differences were found between groups (*t*(37) = − 0.326, p = 0.746, *d*_Cohen_ =  − 0.106), suggesting similar amounts of mean overall head movement in both groups.

### IPS Between Dyad Types

To investigate group differences in interpersonal synchrony, we performed an independent samples t-test comparing ASD+ and ASD− . We found a significant difference of IPS head synchrony between the ASD + and the ASD- group (*M* = 0.061 and *M* = 0.070 respectively, *t*(37) = − 2.068, p = 0.023, one-tailed, *d* = − 0.673). IPS was significantly lower in interviews with ASD+ patients than with ASD− patients not fulfilling diagnostic criteria.

### Differences in Included Self-Report Questionnaires

**Fig. 1:**
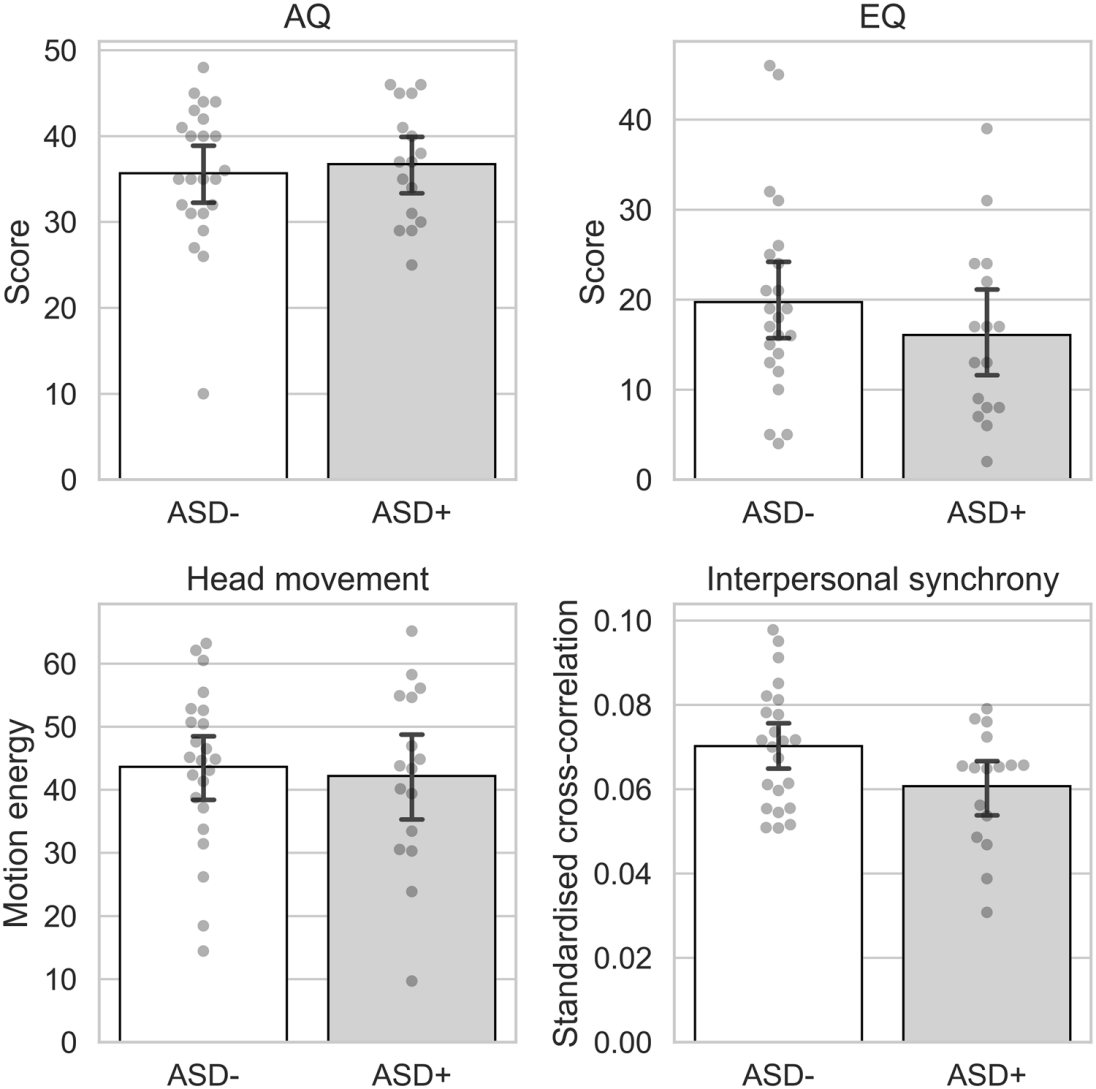
Mean group values for (upper, left) autistic traits, (upper, right) empathy, (lower, left) motion energy in the head ROI, and (lower, right) interpersonal synchrony in the head ROI. Bars represent 95% confidence intervals

We conducted an independent samples t-test for the included standard screening instruments for autism traits (AQ) and empathy (EQ) and subsequently correlated them with the IPS values within groups. We found no significant group differences for both (AQ: *U* = 192, p = 0.830, *d* = 0.043; EQ: *t*(37) = − 1.065, p = 0.294, *d* = − 0.347; see Table 1 and Fig. 1). For the dyspraxia questionnaire (ADC), scores of 4 participants (3 ASD− and 1 ASD+) were missing and imputed with the respective group average. We found no significant group differences (*U* = 239.5, p = 0.116, *d* = 0.302; see Table 1 and Fig. 1) and no significant association with IPS. A correlation table can be found in the supplementary materials.

## Discussion

Autism differential diagnostics in adulthood presents a challenge due to high heterogeneity and comorbidity rates. The complexity of the diagnostic procedures combined with the rising number of individuals seeking diagnostic classification justify the search for observer-independent diagnostic tools (see research priorities published by Autistica, 2016). We tested the potential of IPS as an automatized aid for differential diagnostics in a representative real-world clinical referral population of adults undergoing the current standard diagnostic procedure in two specialized autism outpatient clinics in Germany.

While standard screening tools AQ and EQ could not differentiate between patients later diagnosed with ASD and those not fulfilling diagnostic criteria, we found a significant difference in IPS between groups. This difference was due to social synchrony differences between dyads and not merely due to differences in individual motoric difficulties. Despite high levels of dyspraxia symptoms in the whole patient population, we found no statistically significant association of IPS with dyspraxia symptoms. Our findings of high dyspraxia scores is in accordance with previous evidence of high prevalence of dyspraxia in adults with ASD (Cassidy et al., 2016).

We have previously shown that reduced IPS could be observed in naturalistic conversations between individuals with ASD with either another individual with ASD or non-affected controls (Georgescu et al., 2020). Our current results extend the previous findings by translating the same measure of nonverbal synchrony into clinical practice and showing specificity of reduced IPS for ASD as compared to clinical controls. Arguably our clinical comparison group was not homogeneous with respect to a certain differential diagnosis (e.g., social phobia), but all patients reported social interaction difficulties and were referred by medical consultants for diagnostic clarification due to a suspected ASD. Indeed, the population assessed in the current translational study represents full clinical reality and the most relevant group comparison of individuals with and without ASD within a real referral population to specialized diagnostic centers. Our results therefore support the idea to further pursue the investigation of IPS as a potential diagnostic marker for ASD in a population representative for specialized autism units. Additional comparison between individuals with ASD and several homogeneous samples of patients with differential diagnoses (e.g. social phobia) will be required by future research in order to confirm our finding of specificity of IPS for ASD. Given the rise in computational methodology for translational psychiatric research (Dwyer et al., 2018) and in ASD specifically (Thabtah, 2018), future research should additionally employ machine learning methods to further investigate the potential for IPS in individual diagnosis prediction. First findings point to a promising role of these methods in digitally-assisted diagnostics for ASD in a social interaction context encompassing i.e. facial mimicry (Drimalla et al., 2020) and intrapersonal synchrony (Georgescu et al., 2019).

In this study we found that standard screening tools in adult autism diagnostics might not be equally reliable as IPS in screening for ASD *within* a clinical population. Indeed, patients with schizophrenia have been found to score equally high on the AQ (Kästner et al., 2015), suggesting a high overlap in symptomatology with the autistic phenotype. Also patients with episodic and chronic depression report significantly more autistic traits than healthy controls (Domes et al., 2016). In addition, self-report measures are characterized by a high degree of subjectivity. In contrast to the lack of differentiation of the self-report measures, we found a significant reduction in objectively measured IPS during diagnostic interviews of patients with ASD compared to patients without ASD. Importantly, they were comparable in total head movement measured in the dyad, suggesting that the reduction in IPS was not due to differences in overall movement quantity in interaction partners.

Interestingly, IPS across this sample was not associated with the extent of motor difficulties, suggesting further underlying processes for reduced IPS in ASD. This is in concordance with findings by Fitzpatrick et al. (2017), who investigated synchronization in three different motor tasks in ASD and their relation to motor difficulties. While the extent of motor skills correlated positively for IPS in a rhythmic hand clapping task, it did not for an imitation and a synchrony test battery, leading the authors to the conclusion that the relationship between IPS and motor skills may not be straightforward but instead dependent on the amount of motor timing that is required of the participant (ibid.). Nevertheless, our finding of elevated motor impairments in autistic adults is in agreement with a previous study (Cassidy et al., 2016) and highlights the importance of consideration of motoric problems in clinical care.

### Limitations

Important limitations of this study must be considered when interpreting the results. We recognize that our findings will need to be replicated in a larger sample and with specific homogeneous clinical control samples complementing our results of specificity of reduced IPS for adults with a confirmed ASD diagnosis within a referral population of individuals with a suspicion of ASD. Furthermore, MEA, though frequently used to quantify IPS and being substantially more time-efficient and objective than manual coding, has certain methodological constraints (as discussed before, Georgescu et al., 2020). In particular, while we chose MEA due to our focus on timing of social interaction and easy translation into clinical practice, other methods might be used in the future to complement our findings with abnormalities in spatial movement patterns, such as 3D tracking methods or qualitative variables underlying our findings of reduced head IPS in ASD. It is important to note that sophisticated motion capture technology, while certainly being excellent tools for fine-grained motion tracking, pose specific constraints to a clinical population, are high-cost and may currently not readily be translatable to clinical practice. Lastly, due to the dyadic nature of the output variable IPS, we cannot entirely rule out the possibility that the perceived IPS by the clinician conducting the interview might have influenced the impression formation of the conversational partner. Indeed, previous research has shown that perceived synchrony in a motor-tapping task increases empathy towards a partner (Koehne et al., 2015). Thus, while the diagnostic decision was made by specialized clinical experts not taking part in the data collection and considering multiple anamnestic sources adhering to German diagnostic S3-guidelines for ASD (AWMF, 2016), perceived IPS might have influenced the attitude and degree of understanding of the interviewer towards the patient. This becomes especially crucial within the “double-empathy problem” framework in autism (Milton, 2012), explaining the disconnection between two interaction partners by a lack of understanding for the other. By investigating a measure of interpersonal coordination like IPS, we are essentially moving away from individual deficits alone to include dyadic measures. This is particularly important in autism, as a condition with social difficulties but also in the broader context of “2nd person psychiatry” (Schilbach, 2016).

## Conclusion

In conclusion, we found IPS to significantly differ between individuals with and without ASD within a referral population in two specialized outpatient clinics for autism, while standard autism screening tools did not. Importantly however, we believe we are not introducing a new phenomenological marker, but merely provide an objective quantification for irregularities in temporal coordination between people with autism and their diagnosticians, which an experienced diagnostician recognizes intuitively, and which influences their clinical impression of the patient’s nonverbal communication, quality of interaction and rapport. We argue though that given the pressing need of more economic and reliable diagnostics, we should aim for digitally aided diagnostics, not to substitute but to complement clinical impression making. In any case, more research will be needed to replicate our findings, establish generalizability and translate these basic findings into clinical practice.

## Supplementary Information

Below is the link to the electronic supplementary material.

Supplementary file1 (DOCX 561 kb)

## Data Availability

Neither of the experiments reported in this article was formally preregistered. The datasets generated and/or analyzed during the current study are not publicly available as they are from a clinical sample that did not consent to their data being shared in any form. The raw data are not available to be shared. The data are available from the corresponding author on reasonable request.

## Abbreviations

ASD: Autism spectrum disorder
AQ: Autism quotient
EQ: Empathy quotient
TD: Typically developing
IPS: Interpersonal synchrony
DCD: Developmental coordination disorder
TAS20: Toronto Alexithymia Scale
ADC: Adult dyspraxia questionnaire
BDI: Beck depression inventory
MEA: Motion energy analysis
ROI: Region of interest
