# Supplementary material for: Brief Report: Specificity of Interpersonal Synchrony Deficits to Autism Spectrum Disorder and Its Potential for Digitally Assisted Diagnostics
Source: J Autism Dev Disord. 2021 Jul 31;52(8):3718–26. doi: 10.1007/s10803-021-05194-3 (PMC9296396; doi:10.1007/s10803-021-05194-3)

APPENDIX

| \| **Test of Normality (Shapiro-Wilk)** \| \| \| \| \| \| \| \| \| --- \| --- \| --- \| --- \| --- \| --- \| --- \| --- \| \|  \| \|  \| \| **W** \| \| **p** \| \| \| all_lags_head \|  \| ASD+ \|  \| 0.925 \|  \| 0.200 \|  \| \|  \|  \| ASD- \|  \| 0.948 \|  \| 0.262 \|  \| \| all_lags_body \|  \| ASD+ \|  \| 0.954 \|  \| 0.554 \|  \| \|  \|  \| ASD- \|  \| 0.950 \|  \| 0.293 \|  \| \| all_lags_total \|  \| ASD+ \|  \| 0.957 \|  \| 0.601 \|  \| \|  \|  \| ASD- \|  \| 0.948 \|  \| 0.269 \|  \| \|  \| \| \| \| \| \| \| \| \| Note.  Significant results suggest a deviation from normality. \| \| \| \| \| \| \| \|  IPS for Body and Total ROIs *Note: one-sided testing, based on previous literature   \| **Test of Equality of Variances (Levene's)** \| \| \| \| \| \| \| \| \| --- \| --- \| --- \| --- \| --- \| --- \| --- \| --- \| \|  \| \| **F** \| \| **df** \| \| **p** \| \| \| all_lags_head \|  \| 0.038 \|  \| 1 \|  \| 0.846 \|  \| \| all_lags_body \|  \| 0.015 \|  \| 1 \|  \| 0.904 \|  \| \| all_lags_total \|  \| 1.707 \|  \| 1 \|  \| 0.199 \|  \| \|  \| \| \| \| \| \| \| \|   **Independent Samples T-Test** | | | | | | | | | | | | | | | | | | |
| --- | --- | --- | --- | --- | --- | --- | --- | --- | --- | --- | --- | --- | --- | --- | --- | --- | --- | --- | --- | --- | --- | --- | --- | --- | --- | --- | --- | --- | --- | --- | --- | --- | --- | --- | --- | --- | --- | --- | --- | --- | --- | --- | --- | --- | --- | --- | --- | --- | --- | --- | --- | --- | --- | --- | --- | --- | --- | --- | --- | --- | --- | --- | --- | --- | --- | --- | --- | --- | --- | --- | --- | --- | --- | --- | --- | --- | --- | --- | --- | --- | --- | --- | --- | --- | --- | --- | --- | --- | --- | --- | --- | --- | --- | --- | --- | --- | --- | --- | --- | --- | --- | --- | --- | --- | --- | --- | --- | --- | --- | --- | --- | --- | --- | --- | --- | --- | --- | --- | --- | --- | --- | --- | --- | --- | --- | --- | --- | --- | --- | --- | --- | --- | --- | --- | --- | --- | --- | --- | --- | --- | --- | --- | --- | --- | --- | --- |
|  | | | | | | | | | **t** | | | | **df** | | **p** | | **Cohen's d** | |
| all_lags_head | | | | | | | |  | -2.068 | | |  | 37 |  | 0.023 |  | -0.673 |  |
| all_lags_body | | | | | | | |  | -0.463 | | |  | 37 |  | 0.323 |  | -0.151 |  |
| all_lags_total | | | | | | | |  | -0.959 | | |  | 37 |  | 0.172 |  | -0.312 |  |
|  | | | | | | | | | | | | | | | | | | |
| *Note.*  For all tests, the alternative hypothesis specifies that group *ASD+* is less than group *ASD-* | | | | | | | | | | | | | | | | | | |
| **Group Descriptives** | | | | | | | | | | | | | | | | | | |
|  | | **Group** | | **N** | | **Mean** | | **SD** | | **SE** | |  |  |  |  |  |  |  |
| all_lags_head |  | ASD+ |  | 16 |  | 0.061 |  | 0.014 |  | 0.003 |  |  |  |  |  |  |  |  |
|  |  | ASD- |  | 23 |  | 0.070 |  | 0.014 |  | 0.003 |  |  |  |  |  |  |  |  |
| all_lags_body |  | ASD+ |  | 16 |  | 0.076 |  | 0.014 |  | 0.003 |  |  |  |  |  |  |  |  |
|  |  | ASD- |  | 23 |  | 0.078 |  | 0.014 |  | 0.003 |  |  |  |  |  |  |  |  |
| all_lags_total |  | ASD+ |  | 16 |  | 0.080 |  | 0.012 |  | 0.003 |  |  |  |  |  |  |  |  |
|  |  | ASD- |  | 23 |  | 0.085 |  | 0.015 |  | 0.003 |  |  |  |  |  |  |  |  |
|  | | | | | | | | | | | |  |  |  |  |  |  |  |

#

# Motion energy for Body and Total ROIs

| *Note: mean_rel_move_dyad = total ROI, two-sided   \| **Test of Normality (Shapiro-Wilk)** \| \| \| \| \| \| \| \| \| --- \| --- \| --- \| --- \| --- \| --- \| --- \| --- \| \|  \| \|  \| \| **W** \| \| **p** \| \| \| mean_rel_move_dyad \|  \| ASD+ \|  \| 0.935 \|  \| 0.297 \|  \| \|  \|  \| ASD- \|  \| 0.913 \|  \| 0.048 \|  \| \| mean_rel_move_head \|  \| ASD+ \|  \| 0.969 \|  \| 0.828 \|  \| \|  \|  \| ASD- \|  \| 0.957 \|  \| 0.399 \|  \| \| mean_rel_move_body \|  \| ASD+ \|  \| 0.951 \|  \| 0.503 \|  \| \|  \|  \| ASD- \|  \| 0.935 \|  \| 0.141 \|  \| \|  \| \| \| \| \| \| \| \| \| Note.  Significant results suggest a deviation from normality. \| \| \| \| \| \| \| \|  \| **Test of Equality of Variances (Levene's)** \| \| \| \| \| \| \| \| \| --- \| --- \| --- \| --- \| --- \| --- \| --- \| --- \| \|  \| \| **F** \| \| **df** \| \| **p** \| \| \| mean_rel_move_dyad \|  \| 1.806 \|  \| 1 \|  \| 0.187 \|  \| \| mean_rel_move_head \|  \| 0.301 \|  \| 1 \|  \| 0.587 \|  \| \| mean_rel_move_body \|  \| 1.673 \|  \| 1 \|  \| 0.204 \|  \| \|  \| \| \| \| \| \| \| \|   **Independent Samples T-Test** | | | | | | | | | |
| --- | --- | --- | --- | --- | --- | --- | --- | --- | --- | --- | --- | --- | --- | --- | --- | --- | --- | --- | --- | --- | --- | --- | --- | --- | --- | --- | --- | --- | --- | --- | --- | --- | --- | --- | --- | --- | --- | --- | --- | --- | --- | --- | --- | --- | --- | --- | --- | --- | --- | --- | --- | --- | --- | --- | --- | --- | --- | --- | --- | --- | --- | --- | --- | --- | --- | --- | --- | --- | --- | --- | --- | --- | --- | --- | --- | --- | --- | --- | --- | --- | --- | --- | --- | --- | --- | --- | --- | --- | --- | --- | --- | --- | --- | --- | --- | --- | --- | --- | --- | --- | --- | --- | --- | --- | --- | --- | --- | --- | --- | --- | --- | --- | --- | --- | --- | --- | --- | --- | --- | --- | --- | --- | --- | --- | --- | --- | --- | --- | --- | --- | --- | --- | --- | --- | --- | --- | --- |
|  | | **t** | | **df** | | **p** | | **Cohen's d** | |
| mean_rel_move_dyad |  | -0.112 |  | 37 |  | 0.911 |  | -0.037 |  |
| mean_rel_move_head |  | -0.326 |  | 37 |  | 0.746 |  | -0.106 |  |
| mean_rel_move_body |  | -0.215 |  | 37 |  | 0.831 |  | -0.070 |  |
|  | | | | | | | | | |
| Note.  Student's t-test. | | | | | | | | | |

| **Group Descriptives** | | | | | | | | | | | |
| --- | --- | --- | --- | --- | --- | --- | --- | --- | --- | --- | --- |
|  | | **Group** | | **N** | | **Mean** | | **SD** | | **SE** | |
| mean_rel_move_dyad |  | ASD+ |  | 16 |  | 61.809 |  | 16.759 |  | 4.190 |  |
|  |  | ASD- |  | 23 |  | 62.313 |  | 11.341 |  | 2.365 |  |
| mean_rel_move_head |  | ASD+ |  | 16 |  | 42.214 |  | 14.342 |  | 3.585 |  |
|  |  | ASD- |  | 23 |  | 43.635 |  | 12.698 |  | 2.648 |  |
| mean_rel_move_body |  | ASD+ |  | 16 |  | 50.323 |  | 17.096 |  | 4.274 |  |
|  |  | ASD- |  | 23 |  | 51.317 |  | 11.838 |  | 2.468 |  |
|  | | | | | | | | | | | |

# IPS VS PSEUDO FOR BODY AND TOTAL ROI

*Note: Group. 0 = real IPS, group 1 = pseudo IPS; one-sided testing based on previous literature

| **Test of Normality (Shapiro-Wilk)** | | | | | | | |
| --- | --- | --- | --- | --- | --- | --- | --- |
|  | |  | | **W** | | **p** | |
| all_lags_body |  | 0 |  | 0.985 |  | 0.859 |  |
|  |  | 1 |  | 0.985 |  | < .001 |  |
| all_lags_total |  | 0 |  | 0.987 |  | 0.928 |  |
|  |  | 1 |  | 0.984 |  | < .001 |  |
|  | | | | | | | |
| *Note.*  Significant results suggest a deviation from normality. | | | | | | | |

| **Test of Equality of Variances (Levene's)** | | | | | | | |
| --- | --- | --- | --- | --- | --- | --- | --- |
|  | | **F** | | **df** | | **p** | |
| all_lags_body |  | 0.071 |  | 1 |  | 0.789 |  |
| all_lags_total |  | 0.548 |  | 1 |  | 0.459 |  |
|  | | | | | | | |

| **Independent Samples T-Test** | | | | | | | | | |
| --- | --- | --- | --- | --- | --- | --- | --- | --- | --- |
|  | | **W** | | **df** | | **p** | | **Rank-Biserial Correlation** | |
| all_lags_body |  | 22968.000 |  |  |  | 0.030 |  | 0.178 |  |
| all_lags_total |  | 23130.500 |  |  |  | 0.024 |  | 0.186 |  |
|  | | | | | | | | | |
| *Note.*   For the Mann-Whitney test, effect size is given by the rank biserial correlation. | | | | | | | | | |
| *Note.*  For all tests, the alternative hypothesis specifies that group *0* is greater than group *1* . | | | | | | | | | |
| *Note.*  Mann-Whitney U test. | | | | | | | | | |

| **Group Descriptives** | | | | | | | | | | | |
| --- | --- | --- | --- | --- | --- | --- | --- | --- | --- | --- | --- |
|  | | **Group** | | **N** | | **Mean** | | **SD** | | **SE** | |
| all_lags_body |  | 0 |  | 39 |  | 0.077 |  | 0.013 |  | 0.002 |  |
|  |  | 1 |  | 1000 |  | 0.073 |  | 0.013 |  | 4.188e -4 |  |
| all_lags_total |  | 0 |  | 39 |  | 0.083 |  | 0.014 |  | 0.002 |  |
|  |  | 1 |  | 1000 |  | 0.079 |  | 0.013 |  | 4.157e -4 |  |
|  | | | | | | | | | | | |

# CORRELATIONS BETWEEN QUESTIONNAIRE SCORES AND IPS

|  | Group | AQ (*r*_p_) | EQ (*r*_p_) | ADC (*r*_s_) |
| --- | --- | --- | --- | --- |
| Head IPS | ASD+ | -.323 | .426 | .271 |
|  | ASD- | .080 | -.010 | .055 |
|  | All | -.080 | .198 | .063 |
| *Note.* Association of head interpersonal synchrony with autistic traits (AQ), empathy (EQ) and dyspraxia (ADC) scores. *r*_p_ = Pearson correlation coefficient, *r*_s_ = Spearman correlation coefficient. All *p*s > .05. | | | | |

# ALTERNATIVE VISUALISATION OF FIGURE 1: VIOLIN PLOTS WITH MEDIAN AND DISTRIBUTION QUARTILES

#
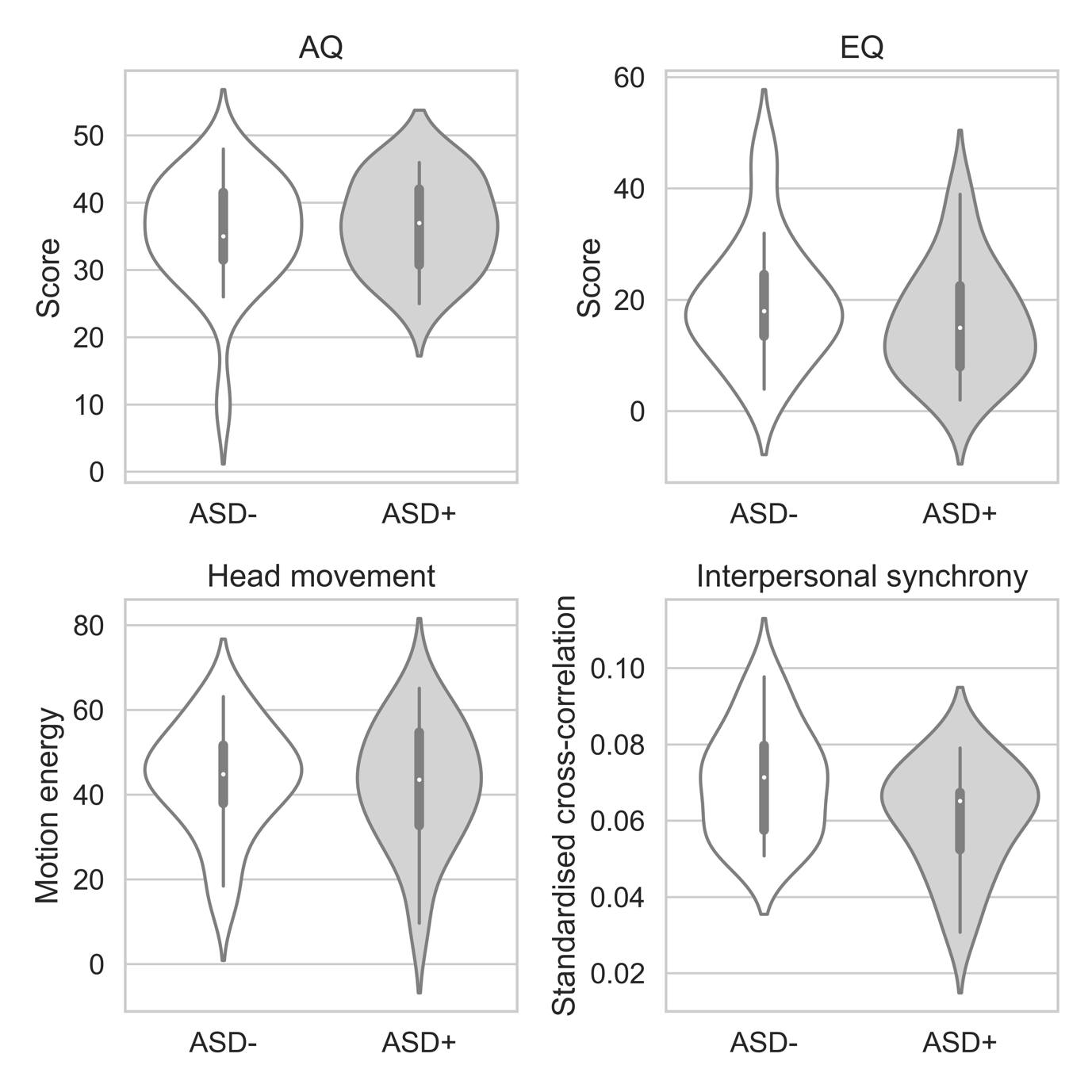


# CORRELATION PLOTS


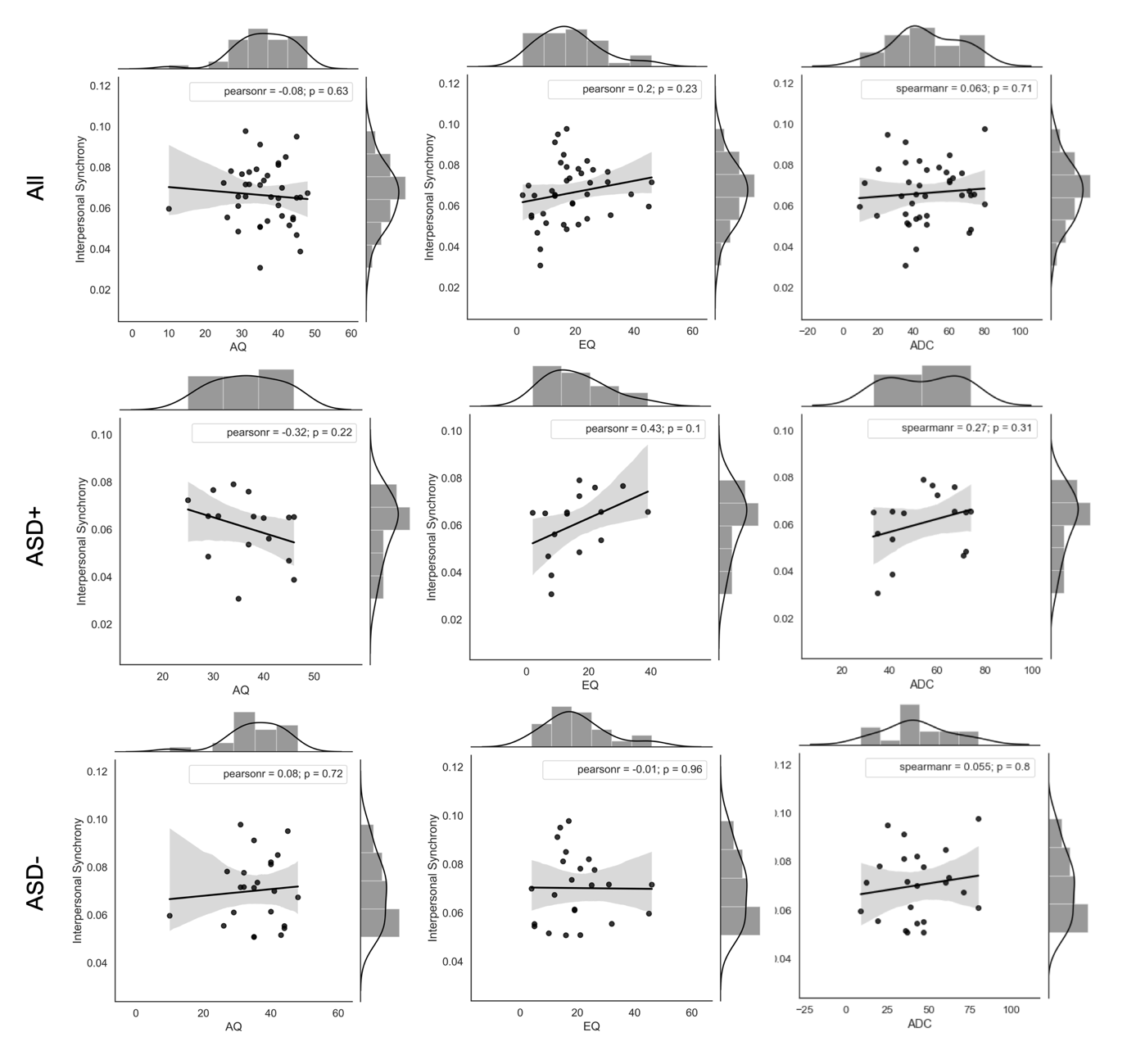

Supplement: Supplementary file 1 — Supplementary file1 (DOCX 561 kb) [file 10803_2021_5194_MOESM1_ESM.docx]
